# Adult mouse hippocampal transcriptome changes associated with long-term behavioral and metabolic effects of gestational air pollution toxicity

**DOI:** 10.1038/s41398-020-00907-1

**Published:** 2020-07-07

**Authors:** Amin Haghani, Richard G. Johnson, Nicholas C. Woodward, Jason I. Feinberg, Kristy Lewis, Christine Ladd-Acosta, Nikoo Safi, Andrew E. Jaffe, Constantinos Sioutas, Hooman Allayee, Daniel B. Campbell, Heather E. Volk, Caleb E. Finch, Todd E. Morgan

**Affiliations:** 1 Leonard Davis School of Gerontology, University of Southern California, Los Angeles, CA USA; 2 Department of Epidemiology, Johns Hopkins Bloomberg School of Public Health, Baltimore, MD USA; 3 Wendy Klag Center for Autism and Developmental Disabilities, Johns Hopkins Bloomberg School of Public Health, Baltimore, MD USA; 4 Department of Pediatrics and Human Development, Michigan State University College of Human Medicine, Grand Rapids, MI USA; 5 Department of Mental Health, Johns Hopkins Bloomberg School of Public Health, Baltimore, MD USA; 6 Lieber Institute of Brain Development, Johns Hopkins Medical Campus, Baltimore, MD USA; 7 Department of Civil and Environmental Engineering, Viterbi School of Engineering, University of Southern California, Los Angeles, CA USA; 8 Department of Preventive Medicine, University of Southern California, Los Angeles, CA USA; 9 Department of Environmental Health and Engineering, Johns Hopkins Bloomberg School of Public Health, Baltimore, MD USA

**Keywords:** Autism spectrum disorders, Molecular neuroscience

## Abstract

Gestational exposure to air pollution increases the risk of autism spectrum disorder and cognitive impairments with unresolved molecular mechanisms. This study exposed C57BL/6J mice throughout gestation to urban-derived nanosized particulate matter (nPM). Young adult male and female offspring were studied for behavioral and metabolic changes using forced swim test, fat gain, glucose tolerance, and hippocampal transcriptome. Gestational nPM exposure caused increased depressive behaviors, decreased neurogenesis in the dentate gyrus, and increased glucose tolerance in adult male offspring. Both sexes gained fat and body weight. Gestational nPM exposure induced 29 differentially expressed genes (DEGs) in adult hippocampus related to cytokine production, IL17a signaling, and dopamine degradation in both sexes. Stratification by sex showed twofold more DEGs in males than females (69 vs 37), as well as male-specific enrichment of DEGs mediating serotonin signaling, endocytosis, Gαi, and cAMP signaling. Gene co-expression analysis (WCGNA) identified a module of 43 genes with divergent responses to nPM between the sexes. Chronic changes in 14 DEGs (e.g., microRNA9-1) were associated with depressive behaviors, adiposity and glucose intolerance. These genes enriched neuroimmune pathways such as HMGB1 and TLR4. Based on cerebral cortex transcriptome data of neonates, we traced the initial nPM responses of HMGB1 pathway. In vitro, mixed glia responded to 24 h nPM with lower HMGB1 protein and increased proinflammatory cytokines. This response was ameliorated by TLR4 knockdown. In sum, we identified transcriptional changes that could be associated with air pollution-mediated behavioral and phenotypic changes. These identified genes merit further mechanistic studies for therapeutic intervention development.

## Introduction

Air pollution impairs global health and causes morbidities throughout the lifespan^1^. Besides children’s lung dysfunctions, developmental exposure brings neurodevelopmental consequences. Gestational exposure to elevated air pollution is associated with low birth weight^2^ and impaired brain myelination^3,4^, and gray matter volume^5^; functional impairments include children’s cognition^3^, behavior^3,6^, and increased risk of autism spectrum disorders^7,8^, and attention deficit hyperactivity disorder^9,10^. Experimental studies validated several of these relationships by exposing rodents during gestation to defined concentrations of air pollution particulate matter PM2.5 or PM0.2. Gestational exposure of rodents can alter adult brain morphology (hypermyelination, ventriculomegaly)^11^, impair memory^12^, and increase depressive symptoms^13^, aggressive behaviors^14^, and post-natal weight gain^15^.

Importantly, epidemiological and experimental studies document sex-specific effects of gestational air pollution exposure. For example, boys were more vulnerable to developmental air pollution exposure for risk of cognitive decline and attention problems^16,17^. A similar male excess was shown for mice exposed gestationally to PM0.2 or diesel exhaust particles for hypermyelination^18^, depressive behavior^13^, and insulin resistance^19^.

The transcriptional processes underlying these effects are relatively unexplored. Only two previous studies documented brain transcriptome responses to gestational air pollution exposure with 70-fold differences in the number of gene responses to air pollution: cerebellar responses to gestational PM2.5 were limited to 11 differentially expressed genes (DEGs) related to inflammation at post-natal day 12^20^. Moreover, gestational exposure to carbon black nanoparticles induced 700 DEG responses in cerebral cortex of adult mice after, including dopamine receptor signaling, inflammation, and growth factors^21^. Because these studies did not examine sex-specific transcriptome responses, we analyzed the hippocampal transcriptome of both male and female adult mice that were gestationally exposed to nanosized urban particulate matter (nPM). The transcriptomic changes were then analyzed in relation to phenotypes including depressive behavior, and systemic metabolic changes.

The findings led us to explore the initial nPM responses in the high mobility group box 1 (HMGB1) pathway in the cerebral cortex of neonates and mixed glial culture. HMGB1 is a multi-functional protein mediator of DNA replication^22^, repair^23,24^, and transcription^25^. Extracellular HMGB1 mediates inflammatory responses by binding to pattern recognition receptors (e.g., TLR4), LPS, IL1B, and others^26^. A persistent change in this pathway is a link between neuroimmune changes and the long-lasting developmental effects of gestational air pollution exposure.

## Methods

### nPM collection

nPM was collected on Teflon filters (20625.4 cm, PTFE, 2 mm pore; Pall Life Sciences) from urban air in Los Angeles, CA, near the CA-110 Freeway using a High-Volume Ultrafine Particle (HVUP) Sampler at 400 L/min from June-August 2016. Filter collected PM0.2 (nPM) was eluted by sonication in deionized water and stored at −20 °C. The aqueous suspension was aerosolized to 300 μg/m^3^ throughout exposure. The re-aerosolized nPM in animal exposure chambers were collected on filters for chemical characterization by high resolution mass spectrometry (SF-ICPMS) and Sievers 900 total organic carbon analyzer^27^. Endotoxin content of the nPM suspension was assessed by a *Limulus* assay (Pierce LAL chromogenic endotoxin, ThermoFisher). For chemical characteristics of nPM, see Supplementary Fig. S1.

### Ethics statement

The Institutional Animal Care and Use Committee at USC approved these experiments (protocol #11992 & 20720). All studies followed the recommendations in the Guide for the Care and Use of Laboratory Animals of the National Institutes of Health.

### Gestational exposure

C57BL/6J mice at 9 weeks of age were obtained from The Jackson Laboratory. Breeding trios (2 females and 1 male) were randomly assigned to two exposure groups: nPM and filtered air. Mice were exposed to filtered air or re-aerosolized nPM (300 μg/m^3^) at beginning of gestational day 2 for 5 h/day (10 am to 3 pm), 3 day/week (Mon, Wed, Fri), for 3 weeks of pregnancy. Pregnant mice were maintained in home cages within the exposure chambers to minimize stress. Filtered air (Ctrl) and nPM exposures were done in parallel in the same room to equalize stress of noise, handling, and exposure time. Exposure stopped with the birth of the first pup. The inhaled PM approximates 27 μg/m^3^ constant exposure, which is experienced in many major cities. The mode of administration of nPM was whole body exposure. Gestational exposure did not alter the litter number. To reduce handling stress and potential maternal neglect, neonates were not weighed, measured, or otherwise disturbed until weaning at 21 days of age. Weaned mice were housed (4–5 per cage) at 25 °C on 12 h light/dark cycles. Offspring from five litters per exposure group were examined as adults. Sample size determined by number of offspring. Treatment groups were blinded during analysis of behavioral testing and histochemistry.

### Body weights and composition

Body weights were recorded every two weeks after weaning. Body compositional analyses were conducted at weeks 6, 12, and 16 using the minSpec NMR machine (Bruker Corporation).

### Forced swim

The forced swim test to assess stress coping strategies^28^ was performed at age 11–13 weeks. Mice were placed in a clear cylindrical water bath at 24–25 °C and recorded 5 min for latency to the first period of immobility and total time immobile.

### Intraperitoneal glucose tolerance test (IPGTT)

At 16 weeks of age, mice underwent IPGTT using standard protocols. Briefly, mice were fasted for 10 h overnight before injection with a bolus (1 mg/g body weight) of glucose (10% wt/vol in sterile water) into the peritoneal cavity. Blood samples were obtained from conscious mice through the tail vein at 0, 15, 30, 60, 90, and 120 min post-injection. Plasma glucose was measured by Freestyle Lite glucometer (Abbott Diabetes Care, Alameda, CA).

### EdU injections

Based on the documented male behavioral bias in gestational response to nPM^13,27^, we chose to examine neurogenesis in male offspring only. Male mice were injected i.p. with 41 μg/g 5-ethynyl-2′-deoxyuridine (EdU) for seven times over 3 days, ending 18 days before tissue collection. All animals were injected with EdU due to the small sample size.

### Tissue collection

At 19 weeks of age, mice were euthanized via cardiac puncture; brains were perfused with 0.9% saline and hemisected. The left hemisphere was fixed overnight in 4% paraformaldehyde in 0.1 M borate buffer (pH 8.5) and cryoprotected in 12% sucrose, plus flash-frozen in isopentane for sagittal sections (30 μm) using a sled microtome fitted with a freezing stage. The hippocampus of the right hemisphere was used for RNA sequencing analysis.

### Immunohistochemistry

Floating sections were permeabilized in Triton-100, blocked in 3% NDS, incubated in primary antibody overnight at 4 °C and in secondary antibody for 4 hours (1:500, ThermoScientific). Primary antibodies were NeuN (1:10,000, ab104224, Abcam), and GFAP (1:1000, ab7260, Abcam). Slides were stained for EdU (Click-iT Plus EdU Alexa Flour 555 Imaging Kit C10639, ThermoFisher Scientific). EdU-positive cells were identified with fluorescence microscopy and colocalized with NeuN and GFAP to determine neurogenesis and astrogenesis. The images were taken at ×20 objectives using a Nikon Eclipse TE300 microscope (Nikone, Melville, NY). Images were analyzed with Image J software.

### RNA sequencing (RNA-seq) of the hippocampus

Five hippocampal samples per group per sex were randomly selected for RNA-seq analysis. The RNA was extracted using QIAGEN RNeasy plus universal protocol (Qiagen #73404). 600 ng RNA was used to prepare RNA-seq libraries using the Illumina RiboZero Gold library preparation kit. The libraries were sequenced by Illumina HiSeq 3000 sequencer at the Lieber Institute for Brain Development. The data preprocessing included quality check by FastQC^29^ and where needed, trimming of the adapter sequences by Trimmomatic^30^. The raw reads were aligned to the mouse reference genome (mm10) using the HISAT2 splice-aware aligner^31^. The overlapping gene alignments were counted using featureCounts version 1.5.0-p3^32^ relative to Gencode version M11 (118,925 transcripts across 48,709 genes, March 2016). Counts were then converted to count per million (CPM) for data visualization and preliminarily assessment in the EdgeR package in R. The data were normalized by the TMM method^33^ and converted to Log2 expression using the Voom package in R for further linear modeling. Differential expression analysis used Empirical Bayes Statistics (eBayes; Limma package). In the first model, sex was considered as a covariate to identify the nPM effects in all groups. Next, the data were stratified by sex, and the nPM effects were studied separately. We set a nominal significance level at *p*-value < 0.005, where no genes were detected at 5% false discovery rate (FDR).

The log2 expression data were further analyzed by weighted gene co-expression analysis (WGCNA). Briefly, the scale-free network was produced by giving the unsigned matrix a soft threshold power. This network was used to calculate the topological overlap and dissimilarity matrices. Following hierarchical clustering, the gene modules were identified by a dynamic tree cut algorithm. Using the singular value decomposition method, the module Eigengene was calculated and tested for association with nPM exposure.

The association of RNA expression of the genes was tested for phenotypic changes as an outcome using linear modeling. Log2 expression of the genes and sex were included in the model as an independent variable. The significance of the association was studied at 5% FDR.

### Ingenuity pathway analysis (IPA)

Identified gene sets were analyzed by IPA (Qiagen) for the enrichment of canonical pathways and candidate upstream regulators. Significance was calculated by right-tailed Fisher’s exact test of the overlap of observed genes with the database.

### Cell culture

Primary mixed glia were cultured from rat cerebral cortex (post-natal day 3; mixed-sex)^34^ at a ratio of astrocytes to microglia, 3:1. For TLR4 knockdown, the cells were transfected with TLR4 siRNA (30 pmol, ThermoFisher Scientific, ID 198667) using lipofectamine RNAiMAX reagent (9 μl/well, ThermoFisher Scientific). After 48 h of transfection, the cells were washed with PBS, and then treated with 10 μg nPM/ml media for 24 h, which did not induce cellular death assessed by LIVE/DEAD cell viability assay (ThermoFisher Scientific) (Supplementary Fig. S2).

### Protein analysis

Total protein was extracted by homogenization in 1× RIPA buffer supplemented with 1 mM Na_3_VO_2_, 1 mM phenylmethylsulfonyl fluoride (PMSF), 10 mM NaF, phosphatase inhibitor cocktail (Sigma), and Complete Mini EDTA-free Protease Inhibitor Cocktail Tablet (Roche). Lysate supernatant was obtained by centrifugation 10,000×*g*/10 min; protein concentration was estimated by Bradford assay. HMGB1 protein was detected by western blot using anti-HMGB1 (1:10,000, Abcam, ab18256) primary antibody. Inflammatory cytokines were analyzed by V-plex proinflammatory panel 2 immunoassays (Mesoscale Diagnostics, Rockville, MD).

### Statistical analysis

RNA-seq data were analyzed in Rstudio as described in each section. The mean differences were analyzed by analysis of variance (ANOVA), followed by pairwise comparison and controlling for multiple test correction at 5% FDR. ANOVA used GraphPad Prism v.7.

## Results

### Gestational exposure to nPM caused transcriptomic changes in the adult hippocampus

**Fig. 1:**
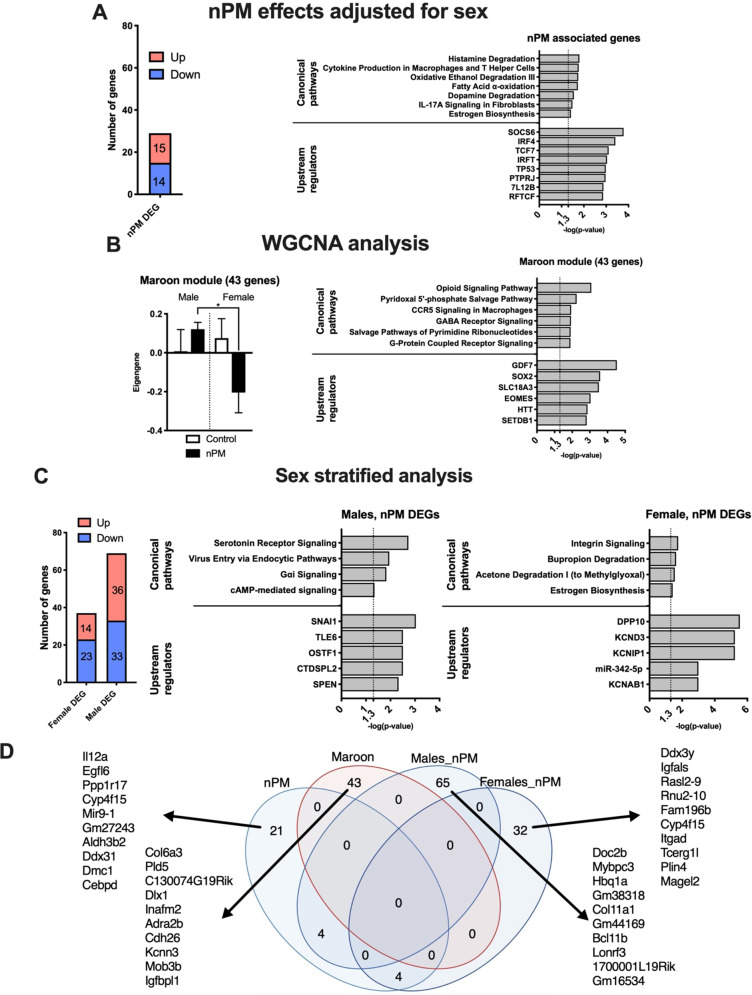
Gestational exposure to nPM caused long-lasting hippocampal transcriptional changes. **a** Number of differentially expressed genes (DEGs) and Ingenuity Pathway Analysis of the gene set in the hippocampus of 19-weeks-old mice gestationally exposed to nPM. The nPM effects were studied at *p* < 0.005 after adjustment for sex differences. **b** WGCNA identified one module associated with nPM. **c** Sex-stratified differential expression analysis of nPM effects in the hippocampus. **d** Venn diagram of the identified gene sets. The top ten genes of each analysis are listed on the figure. *N* = 5/group/sex.

A total of 29 nPM differentially expressed genes (DEGs) (15 upregulated, 14 downregulated) were detected in the hippocampus at 19 weeks of age; both sexes were combined after controlling for sex differences at *p* < 0.005 significance (Fig. 1a). No DEGs were detected at the 5% FDR rate. The identified genes included immune functions (IL17A signaling); metabolism of estrogen, fatty acids, and neurotransmitters (dopamine, histamine). The upstream regulators of these changes include Socs6 (suppressor of cytokine signaling 6, feedback regulator of cytokine signal transduction), IRF4 (interferon regulatory factor 4, interferon-gamma signaling), and P53 (TP53, tumor suppressor gene).

Weighted gene co-expression analysis (WGCNA) identified a module of 43 genes with divergent sex responses to nPM (Fig. 1b). This module involved some nervous system pathways (e.g., opioid and GABA receptors), chemotaxis (e.g., G-coupled receptor and CCR5 signaling), and metabolism (e.g., pyridoxal 5′-phosphate salvage pathway). The regulators of this module include GDF7 (growth differential factor 7, a secreted ligand of TGFB family), and SOX2 (SRY-Box transcriptional factor 2, a mediator of brain stem cell maintenance).

Sex-specific effects of gestational nPM exposure were identified by data stratification. Males had almost twofold more DEGs (69 vs. 37) than females (*p* < 0.005, Fig. 1c). Male DEGs were involved in G-coupled protein receptor signaling (e.g., serotonin receptor, and Gai), endocytosis, and cAMP-mediated signaling. In contrast, female DEGs were related to integrin signaling and estrogen biosynthesis. Top potential regulators of these DEGs were zinc finger protein SNAI1 in males and dipeptidyl peptidase like 10 (DPP10) in females.

The four identified gene sets had low overlap (Fig. 1e). Thus, each statistical approach captured a specific aspect of nPM effects, and altered expression of 153 genes in a sex-specific manner. These genes were further analyzed for association with phenotypic changes.

### Adult brain, behavioral, and systemic responses to gestational nPM exposure

**Fig. 2:**
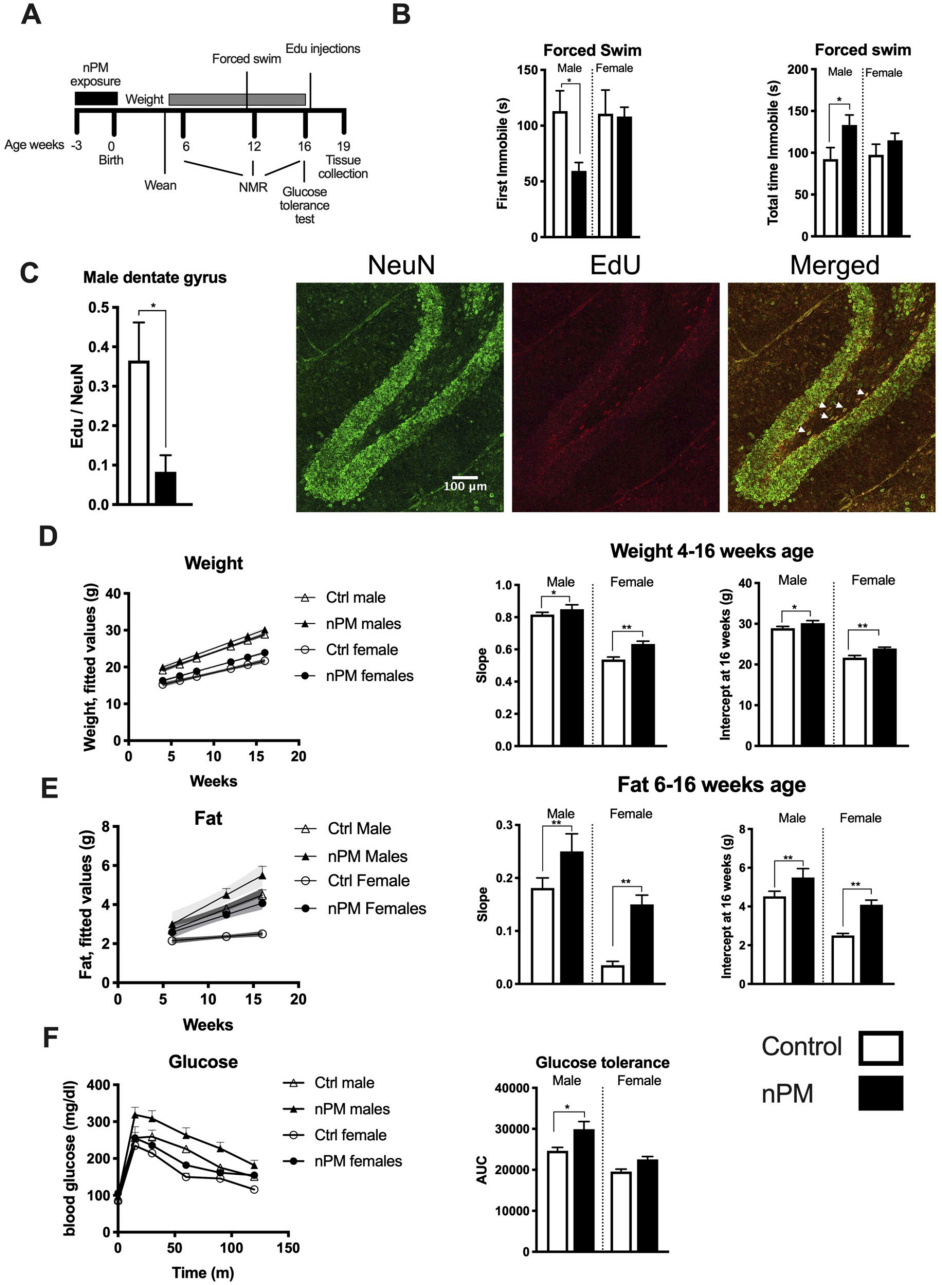
Gestational exposure to nPM caused long-lasting behavioral and systemic changes in young adult mice. **a** Experimental schedule. Following gestational exposure, assessments started after weaning in week 4. NMR: nuclear magnetic resonance. **b** Forced swim test evoked depressive behaviors only in male young adult mice. **c** Neurogenesis was decreased by 70% in the dentate gyrus of male mice (*t*-test). Representative images of NeuN/Edu colocalization in dentate gyrus. Gestational exposure to nPM cause **d** weight and **e** fat gain during development and adulthood of both sexes. The slope and intercept at week 16 of weight and fat were calculated by the mixed effect model including the random effects of the mice in each group. **f** Glucose tolerance test showed increased insulin resistance in male mice after gestational exposure to nPM. Statistical analysis for outcomes with four groups was done by ANOVA with multiple comparisons at a 5% FDR rate. *p < 0.05, **p < 0.01. *N* = 7–17/group/sex.

Depressive behavior of adult offspring was assessed by the forced swim assay at 11–13 weeks of age (Fig. 2a). Only the nPM exposed males showed depressive behavior, with ~50% shorter time to first immobility and ~50% longer total time of immobility than females (Fig. 2b). Neurogenesis was decreased 80% by gestational nPM (EdU+NeuN+) in the dentate gyrus of young adult males at 19 weeks of age (Fig. 2c). Astrogenesis (Edu+GFAP+) was not altered (data not shown).

Both sexes showed increased post-natal growth (Fig. 2d) and adiposity (Fig. 2e) from gestational nPM. Although females had lower baseline weight and fat than males, gestational nPM exposure caused greater weight and fat gain in females (weight: 10% F, 4% M; fat: 60% F, 20% M). Only male mice had increased insulin resistance (20%) from gestational nPM (Fig. 2f).

### Hippocampal transcriptional changes associated with phenotypic changes

**Fig. 3:**
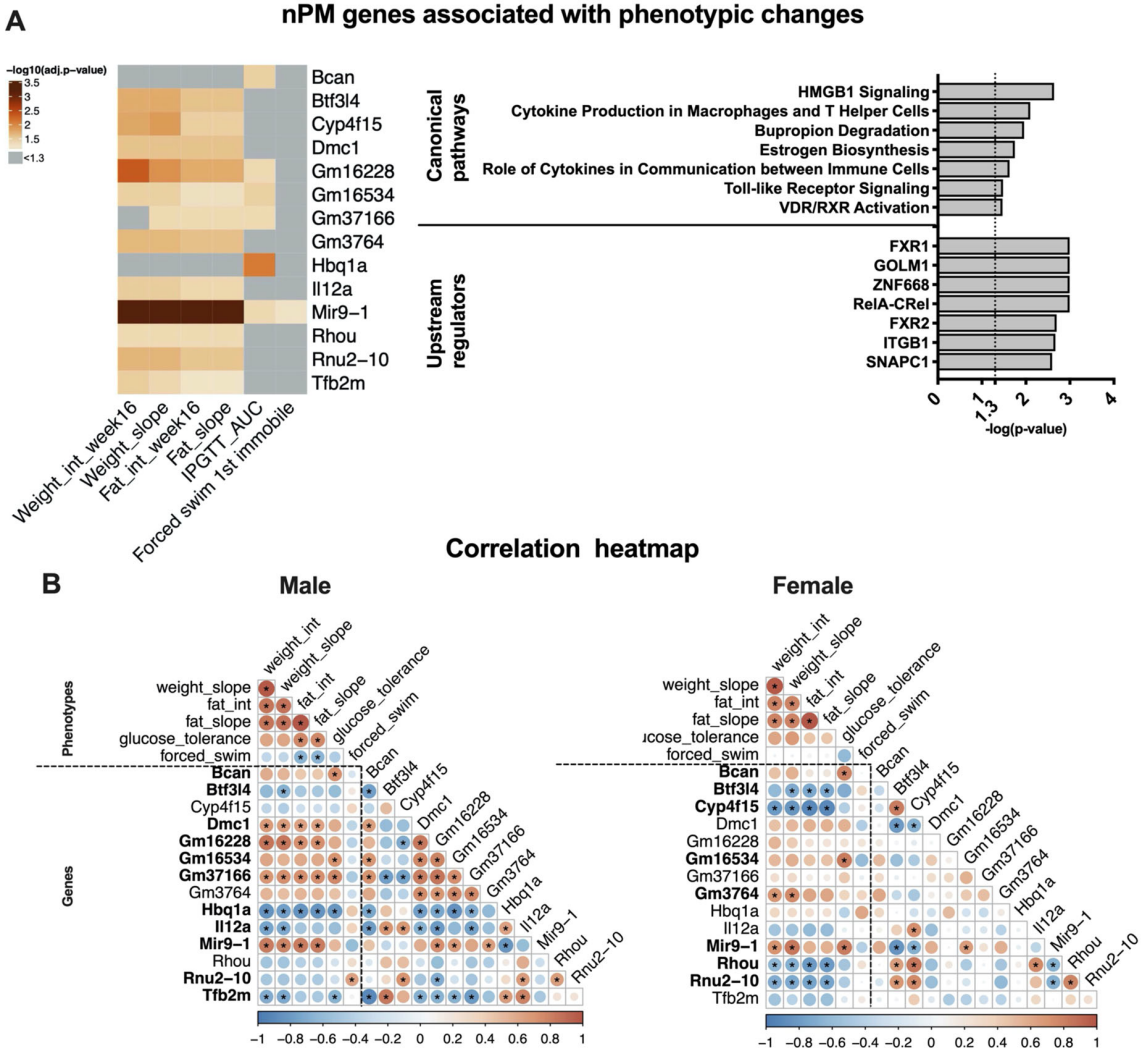
Hippocampal transcriptional changes are associated with long-lasting phenotyping effects of gestational nPM exposure. **a** A subset of 14/153 nPM DEGs was associated with different phenotypes in young adult male and female mice. The models were adjusted for sex differences. The heatmap represents the −log10 (FDR adjusted *p*-values) of the associations. **b** Pearson correlation heatmaps of the measured phenotypes and gene expression changes in male and female mice. *p < 0.05. Bolding indicates at least one significant correlation of gene expression with a phenotype.

The identified 153 responding genes (based on WGCNA and differential expression analysis of total and stratified data) were evaluated for possible associations with phenotypic changes at a 5% FDR rate, using linear modeling and sex as a covariate. Only the level of microRNA9-1 (miR9-1) was associated with all phenotypic changes including the forced swim effect (Fig. 3a).

Glucose intolerance was associated with *Brevican* (Bcan chondroitin sulfate/dermatan sulfate metabolism) and *Hbq1a* (hemoglobin subunit theta 1, iron and oxygen-binding). Weight and fat gain were associated with a shared set of genes including *Il12a* (atypical autism), and *Rhou* (Ras homology family member U, innate immunity). These two genes are parts of the high mobility group box 1 (HMGB1) signaling pathway.

IPA analysis of the 14 identified genes associated with the phenotypic changes enriched immune pathways such as HMGB1 and TLR4 pathways (Fig. 3a). Upstream regulators of these changes included NFKB complex (RELA-CREL), a transcriptional factor of inflammation.

Phenotypes and gene expression changes showed a sex-specific correlation (Fig. 3b; Supplementary Fig. S3). Although weight and fat were correlated in both sexes, the forced swim and glucose tolerance were correlated with fat gain only in males. A subset of 11 genes in males and 8 in females was correlated with all phenotypes (bold in Fig. 3b). Examples of genes with sex-specific phenotypic correlation with changes include *Hbq1a* in males and *Cyp4f15* in females.

### HMGB1 signaling is targeted by nPM

**Fig. 4:**
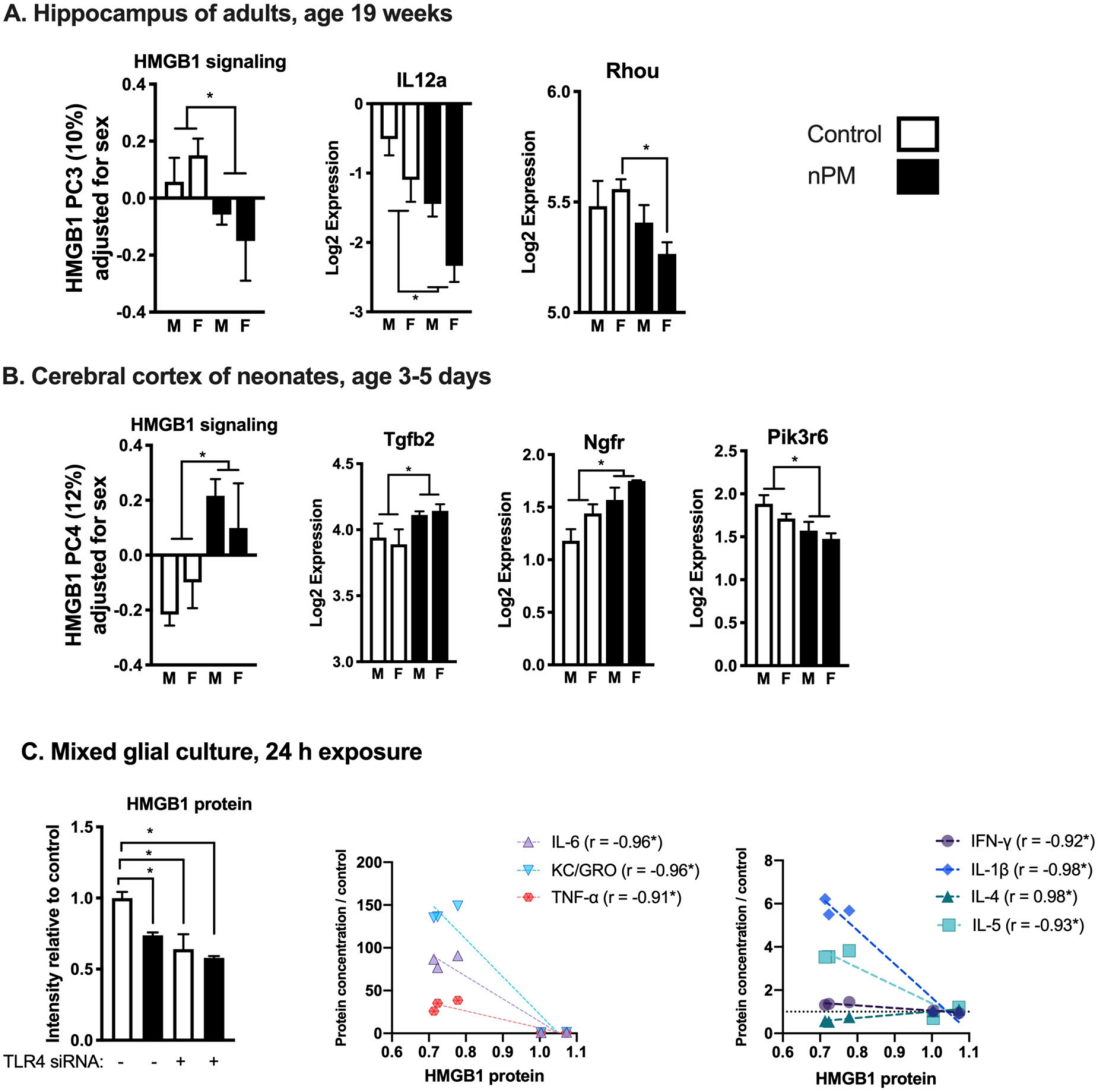
Gestational exposure to nPM alters HMGB1 signaling from early development into adulthood. **a** Principal component analysis and representative changes in 158 genes from HMGB1 signaling pathway in the hippocampus of young adult mice gestationally exposed to nPM. **b** Changes in HMGB1 signaling and some representative genes in the cerebral cortex of neonates that are gestationally exposed to nPM. Principal component analysis of 158 genes from the HMGB1 signaling pathway showed nPM is associated with changes in PC4 of the neonate cerebral cortex. **c** HMGB1 is among the initial responses in rat mixed glial culture after exposure to 10 μg/ml nPM for 24 h. HMGB1 response was TLR4 dependent, shown by siRNA knockdown (*N* = 3 per group, representative of two independent experiments). *FDR adjusted *p*-value < 0.05. The decrease of HMGB1 protein was correlated with the increase of inflammatory cytokines: (IL6, KC/GRO, TNFα, IL1ß, IL5) and decrease of IL4. The correlation analysis did not include groups with TLR4 knockdown. *p < 0.05.

We further examined the HMGB1 pathway as the top canonical pathway that was associated with the chronic phenotypic changes of gestational nPM exposure. HMGB1 signaling is upstream of TLR4-mediated inflammation, and thus includes many genes in the TLR4 signaling pathway. By principal component analysis (PCA) of 158 genes in HMGB1 signaling, gestational nPM exposure was negatively associated with HMGB1 PC3, which explained 10% of expression variance in this pathway (Fig. 4a). Genes downregulated by gestational nPM exposure included *Il12a* (>−200%) and *Rhou* (−100% in females).

To further analyze responses of HMGB1 pathway to gestational nPM exposure, we used another RNA-seq data set (NCBI Geo, GSE142453) generated from the cerebral cortex of the neonates at age 3–5 post-natal days that were siblings of the young adult offspring described in this report. In neonates, exposure to nPM was associated with changes in the cerebral cortex HMGB1 PC4 that explained 12% of the variance in the expression of these 158 genes (Fig. 4b). Gene responding to nPM neonates included *Tgfb2*, +20% (development and immunity); *Ngfr*, +50% (nerve growth factor receptor); and *Pik3r6*, −50% (phosphoinositide-3-kinase gamma adapter, inflammation).

As an in vitro model, we examined responses of the HMGB1 protein in mixed glia (Fig. 4c). Exposure to 10 μg/ml nPM for 24 h, caused 25% decrease in HMGB1 protein. The decrease of HMGB1 protein was correlated with increase of proinflammatory cytokines (KC/GRO, 150 -fold, IL6, 100- fold, TNFA, 25-fold, IL1B, 6-fold, IL5, 4-fold), and 50% decrease of IL4 (an anti-inflammatory cytokine).

Previously, we showed that TLR4 knockdown partially reduces proinflammatory responses of nPM in mixed glia^34^. These further data show that TLR4 knockdown decreased the basal HMGB1 levels, and attenuated nPM response (Fig. 4c), which further links HMGB1 to proinflammatory responses of nPM.

## Discussion

Novel hippocampal transcriptomic responses were identified for gestational air pollution exposure in a mouse model. The changes included immune responses and metabolic pathways related to biosynthesis of dopamine, estrogen, and fatty acid oxidation. These differences paralleled the male excess in phenotypic changes, including depressive behavior and insulin resistance resulting from gestational nPM exposure. We identified a set of genes mainly related to immune responses (e.g., HMGB1 signaling) that were associated with behavioral and metabolic changes. Further analysis revealed that HMGB1 signaling is among the initial response to nPM in the cerebral cortex of neonates PND 3–5 and mixed glial culture.

Gestational nPM affected 29 DEGs related to immune responses, including IL17a signaling and dopamine clearance in the hippocampus of 19-week-old adults. These pathways are shared with transcriptional changes at earlier ages in prior reports^20,21^. Moreover, dopaminergic and inflammatory changes are among adult effects of gestational exposure to PM0.2, PM2.5, and diesel exhaust PM0.2 in mice or rat offspring^12,14,35,36^. Our study extended these findings by identifying new transcriptomic changes with potential upstream regulators, e.g., IRF4 and TP53, for future analysis. These data further support the role of IL17A signaling as a link between ASD to gestational air pollution exposure. In a mouse model for ASD, social behavioral deficits were rescued by delivery of IL17A, but not other cytokines, to primary somatosensory cortex dysgranular zone^37^. In another study, continuous administration of IL17A throughout pregnancy led to a decreased cortical size, neocortical glial density, and a male-specific social approach deficit in adulthood^38^. Together, these data suggest an expanding role of IL17A signaling in the effects of air pollution on brain development and social behaviors.

Epidemiological and experimental studies concur in sex differences of gestational exposure to air pollution. The present study showed a male excess response in depressive behaviors, glucose dysregulation and DEG changes. The baseline sex differences in oxidative stress genes during embryonic and neonatal development may contribute to these divergent responses. For example, several genes related to xenobiotic metabolism (e.g., NRF2 components) are specifically suppressed in male late stage embryos (gestational day 19)^39^. While females have higher expression of some detoxifying enzymes at post-natal day 7.

Male-specific transcriptional responses of nPM included serotonin receptor signaling. Gestational exposure to diesel exhaust PM0.2 was associated with decreased serotonin in nucleus accumbens, amygdala, and hypothalamus, increased dopamine in prefrontal cortex and nucleus accumbens, and increased social isolation-induced territorial aggressive behavior in male mice at 12 weeks of age^14^. We observed male-specific depressive behavior, but did not assess territorial aggressive behavior. Selective serotonin reuptake inhibitors (e.g., SSRIs, SNRIs) are among the current antidepressant drugs in children and adolescents^40^. Thus, changes in serotonin receptor signaling may underlie the depressive behaviors in gestationally exposed mice.

A module of 43 genes showed divergent nPM responses by sex. These genes had functions in some neuronal pathways (e.g., opioid, GABA receptor, and G-coupled receptor signaling), and immune responses such as CCR5 signaling. Sex-specific responses of nPM in these pathways were unresolved in prior studies. EOMES, known as T-box brain protein 2 (TBR2), is a potential regulator of this gene module. The TBR2 protein is transiently expressed during embryonic subventricular zone neurogenesis: it regulates neuronal development including differentiation of upper cortical layer of neurons and also neurogenesis processes in adult dentate gyrus^41^. Developmental effects of nPM on EOMES/TBR2 might contribute to deficits of adulthood neurogenesis observed in males of the present study. Although logistics did not allow us to study females, another study of both sexes showed a male excess of neurogenesis after exposure to diesel exhaust PM0.2 during adulthood^42^. The sex-specific changes in EOMES/TBR2 from developmental exposure to nPM merit further study.

Systemic metabolic changes (e.g., weight and fat gain, and male-specific glucose dysregulation) were among the effects of gestational exposure to nPM. These results extended prior findings by us^19^ and others^15^. Gestation to adulthood exposure of mice to nPM caused male-specific increased food intake, gain in weight and fat, adiposity, glucose intolerance, and changes in expression of several hypothalamic neuropeptides and insulin receptors in adipose tissue^19^. Exposure to air pollution during adulthood alone can also impair glucose tolerance^43–45^, and fat gain^43^. The anti-inflammatory drug (IMD-0354, IKK2 inhibitor) given directly by intra-cerebroventricular administration in adult mice attenuated the observed glucose intolerance and fat gain^43,45^. This observation highlights the underlying role of hypothalamic neuroinflammation in the metabolic changes^43^. The present study identified a set of 14 genes in hippocampus that were associated with these systemic changes. The assessed phenotypes, including the depressive behavior, were inter-correlated, suggesting a shared regulatory mechanism.

*MicroRNA9-1* (*miR9-1*) was the only gene that positively correlated with both depressive behaviors and systemic metabolic changes in male and female mice. *miR9-1* is highly expressed in developing and adult vertebrate brain^46^, and regulates a large gene network involved in proliferation and differentiation of neural progenitor cell population in both embryo^47^ and adult^48^. Changes in *miR9-1* are reported in Alzheimer^49^, Huntington^50^, ALS^51^, Parkinson^52^, and several cancers including glioblastoma^53^. Moreover, *miR9-1* has sex-specific responses. Obese pigs had increased *miR9-1* in subcutaneous adipose tissue of both sexes, whereas in the liver, only males had increased miR9-1^54^. *miR9-1* also regulates several key transcriptional factors, such NF-κB1^55,56^, which is among the initial responses of nPM both in vitro and in vivo^34^.

HMGB1 signaling in the hippocampus was associated with nPM-mediated phenotypic changes. This pathway was also affected in the cerebral cortex of neonates, which exhibited downstream effects of upregulation in *Tgfb2*, *Ngfr*, and downregulation of *Pi3kg*. Notably, PI3KG was identified as a key regulator of IL17 cytokine production in both mouse and cell models^57^. As discussed earlier, IL17 signaling is a novel link between neuro-immunity and social behaviors in the autism spectrum disorders^37^. HMGB1 is a damage-associated protein that reacts with some pattern recognition receptors (e.g., TLR4) and initiates a wide range of inflammatory responses. TLR4 signaling is implicated in both gestational air pollution exposure and autism spectrum disorder^58–60^. In mixed glia, nPM induced a decline in intracellular HMGB1 protein and an increase of proinflammatory cytokines. TLR4 knockdown reduced the HMGB1 baseline, and eliminated the nPM HMGB1 and cytokine response^34^. TLR4 knockdown mouse also had a lower level of HMGB1 protein and inflammatory responses^61^. Thus, our study suggests that acute and chronic changes in HMGB1 and TLR4 signaling might underlie the systemic and behavioral effects of gestational exposure to nPM.

It is unknown how gestational exposure to air pollution causes long-lasting neurodevelopmental changes in offspring. Studies of several populations show that maternal exposure to air pollution affects placental weight and function^62,63^, causes systemic inflammation^64^, and alters cord blood insulin^65^. Air pollution has synergistic effects with maternal stress^66^ and cigarette smoke^67^, which suggest shared mechanisms of action^68^. It is also possible that PM is distributed to the fetus depending on the gestational stage, species, and placental structure^69^.

Regardless of the route of toxicity, interventions are needed to attenuate, or even prevent, the long-term damage. Recent rodent studies suggest modest protective effects against air pollution toxicity for some drugs and nutrient supplements (e.g., ascorbic acid^21^, pioglitazone^42^, probiotics^70^, IMD-0354^43^, and omega-3 fatty acids^71^). For example, 25% of the cerebral cortex DEGs of gestational CNP (carbon black nanoparticles) exposure was prevented by intraperitoneal injection of ascorbic acid in pregnant dams on days 5 and 9 of gestation^21^. These findings implicate oxidative stress and other acute responses in CNP toxicity^21^. Our study identified several phenotypic-specific targets (e.g., HMGB1, *miR9*, and IL17) for intervention studies.

Chemical and physical characteristics of PM warrant more attention in interpreting the toxicity findings. The current studies used nPM, a subfraction of PM0.2 that lacks polycyclic aromatic hydrocarbons and has lower levels of bulk transition metals. Although gestational exposure to nPM and total PM0.2 caused equivalent depressive behaviors in adult offspring, only total PM0.2 caused decreased glutamatergic mRNAs in their cerebral cortex, which may be attributed to PAHs^27^. The current study was on nPM, thus could lack some of the glutamatergic effects of gestational air pollution exposure. We chemically characterized the nPM used here for comparison with future studies. Unfortunately, chemical composition of the experimental PM is often not available in air pollution neurotoxicity research, which limits the reproducibility findings by different researchers.

In sum, our study identified several new potential mechanisms of gestational air pollution toxicity and the gene pathways associated with adult behavior and metabolic changes. These genes should be examined for gene–environment interactions of air pollution contributions to neurodevelopmental disorders.

## Supplementary information

Supplementary Data

## Data Availability

Data are publicly available at GEO NCBI (GSE147842).
