# Supplementary material for: Adult mouse hippocampal transcriptome changes associated with long-term behavioral and metabolic effects of gestational air pollution toxicity
Source: Transl Psychiatry. 2020 Jul 7;10:218. doi: 10.1038/s41398-020-00907-1 (PMC7341755; doi:10.1038/s41398-020-00907-1)
Supplement: Supplementary file 1 — Supplementary Data [file 41398_2020_907_MOESM1_ESM.docx]

**Supplementary Data**

## **Adult mouse hippocampal transcriptome changes associated with long-term behavioral and metabolic effects of gestational air pollution toxicity**

**Running title: Adult mouse hippocampal transcriptome and phenotypic effects of gestational air pollution exposure**

**Authors:** Amin Haghani^1#^, Richard G. Johnson^1#^, Nicholas C. Woodward^1^, Jason I. Feinberg^2,3^, Kristy Lewis^4^, Christine Ladd-Acosta^3,5^, Nikoo Safi^1^, Andrew E. Jaffe^6^, Constantinos Sioutas^7^, Hooman Allayee^8^, Daniel B. Campbell^4&^, Heather E. Volk^3,5,9&^, Caleb E. Finch^1&^, Todd E. Morgan^1&^**^*^**

1, Leonard Davis School of Gerontology, University of Southern California, Los Angeles, CA.

2, Department of Epidemiology, Johns Hopkins Bloomberg School of Public Health, Baltimore, MD.

3, Wendy Klag Center for Autism and Developmental Disabilities, Johns Hopkins Bloomberg School of Public Health, Baltimore, MD.

4, Department of Pediatrics and Human Development, Michigan State University College of Human Medicine, Grand Rapids, MI.

5, Department of Mental Health, Johns Hopkins Bloomberg School of Public Health, Baltimore, MD.

6, Lieber Institute of Brain Development, Johns Hopkins Medical Campus, Baltimore, MD.

7, Department of Civil and Environmental Engineering, Viterbi School of Engineering, University of Southern California, Los Angeles, CA.

8, Department of Preventive Medicine, University of Southern California, Los Angeles, CA.

9, Department of Environmental Health and Engineering, Johns Hopkins Bloomberg School of Public Health, Baltimore, MD.

# Co-first authors

& Contributed equally

*Corresponding author. Email: [temorgan@usc.edu](mailto:cefinch@usc.edu)


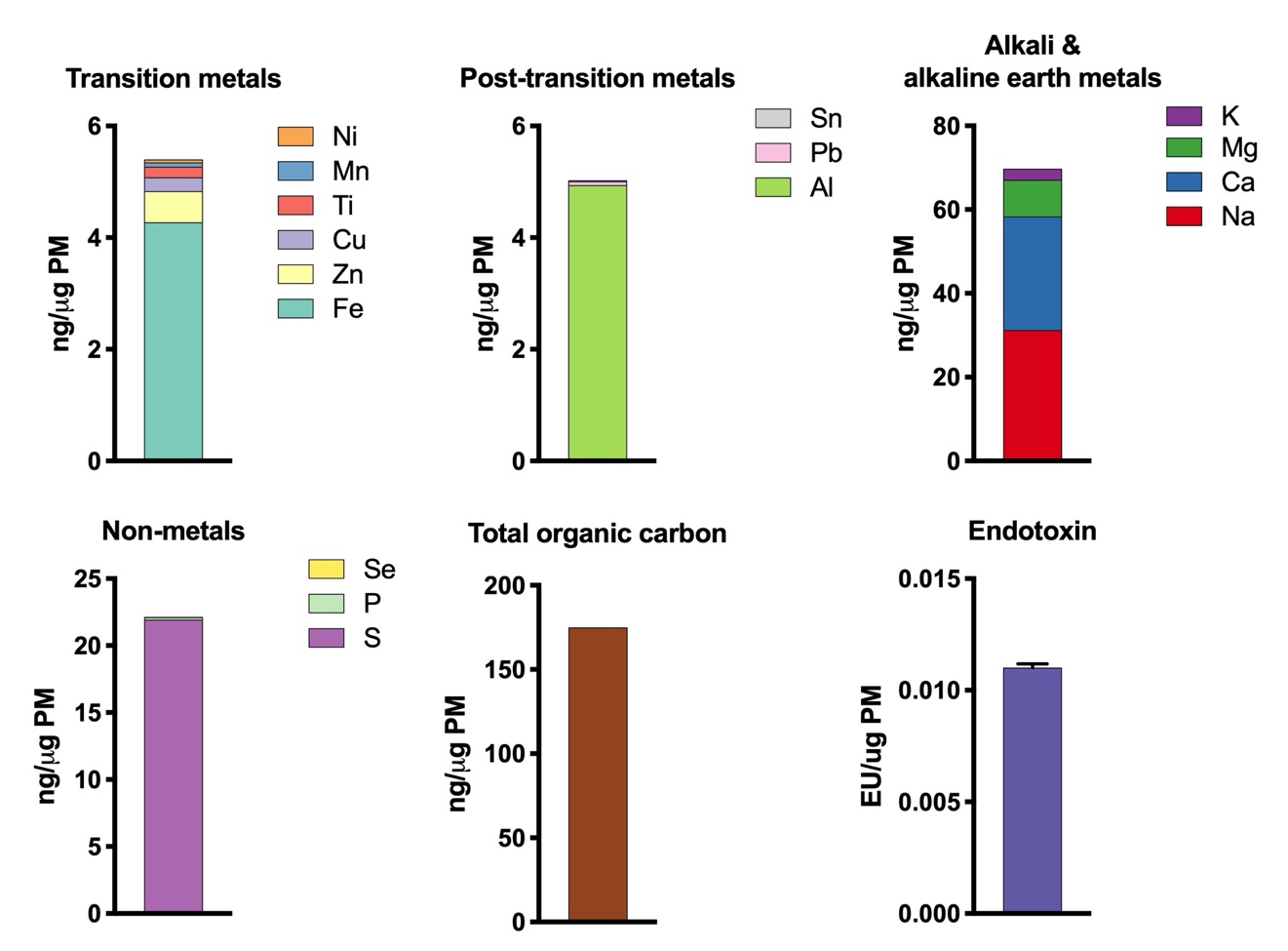


Figure S1. Chemical characterization of nPM samples used in the study.

Figure S2. Cellular toxicity of 10 μg/ml nPM in mixed glial culture after 24 h treatment. The cells were transfected with 30 pmol TLR4 siRNA or scrambled siRNA for 48 h, washed with PBS, then treated with nPM. Identical media changes were performed with the control, non-transfected cells.


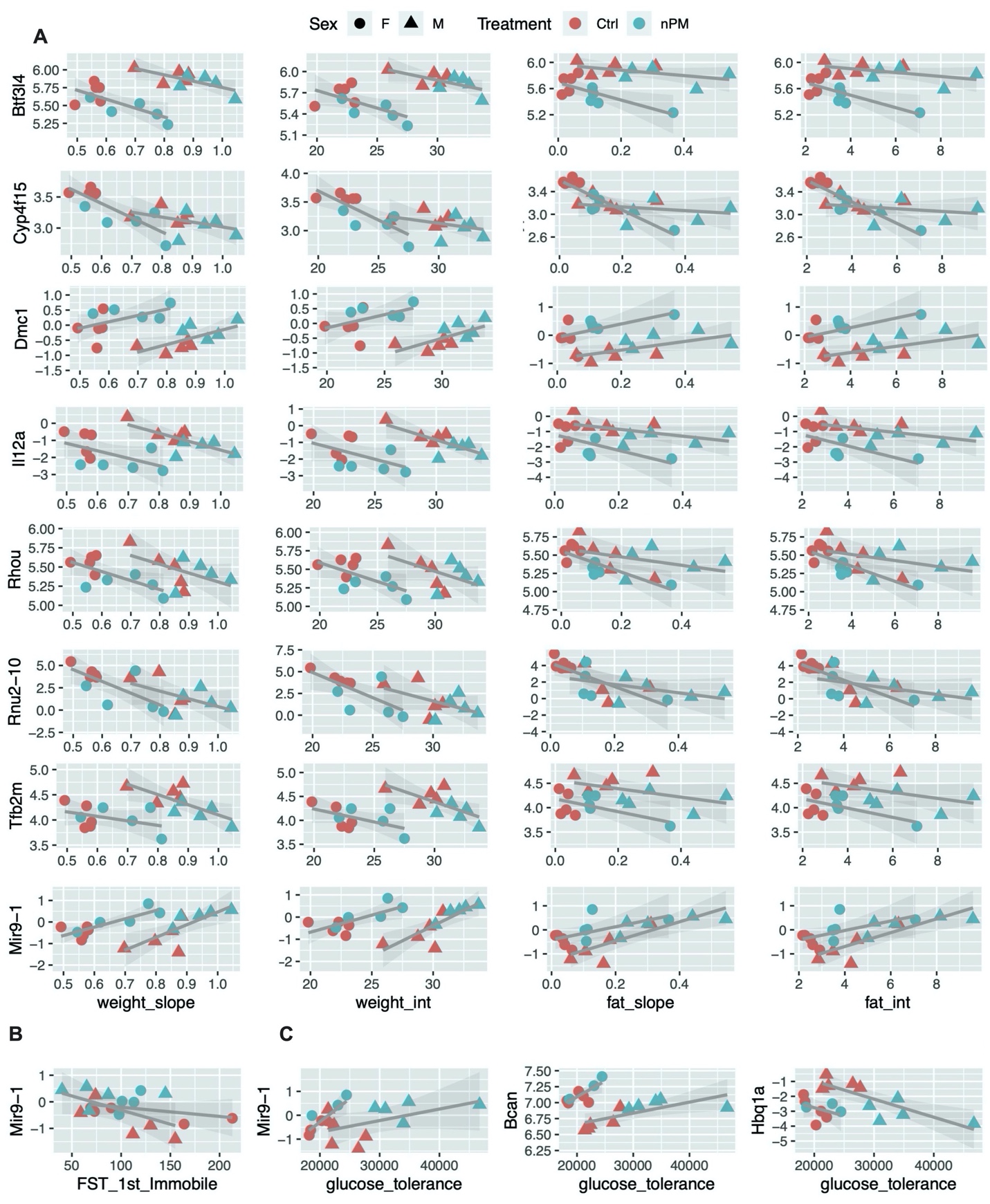


Figure S2. Gene expression associations with depressive behavior and systemic metabolic changes.
